# Supplementary material for: Fragaria ananassa DAM4 expression correlates with vegetative growth during semi-dormancy breaking
Source: Planta. 2025 Aug 17;262(4):84. doi: 10.1007/s00425-025-04799-7 (PMC12358334; doi:10.1007/s00425-025-04799-7)

# Supplement 1

**SON-T, spectrum.**


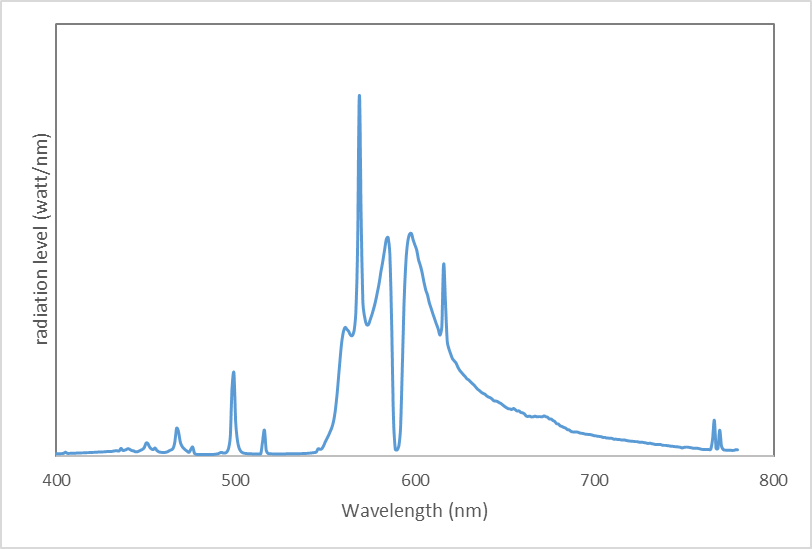


# Supplement 2

**GreenPower LED Flowering Lamp Gen. 2.1 (DRWFR), spectrum.**


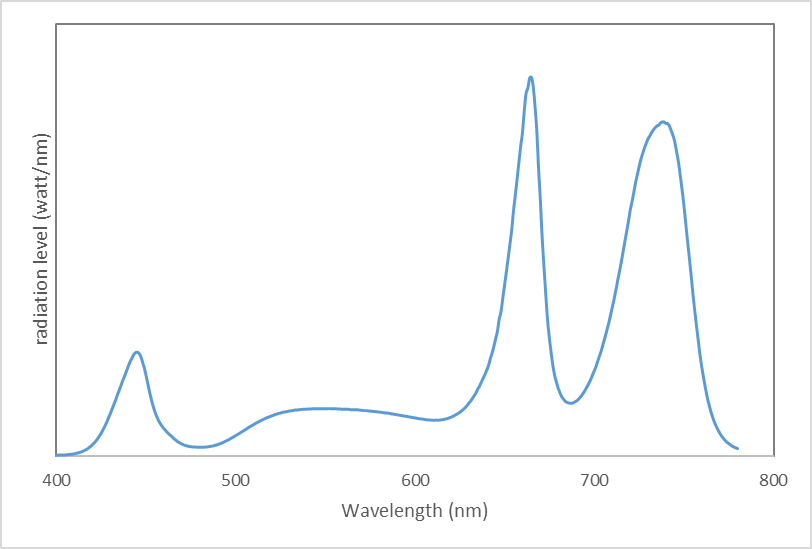


# Supplement 3

**Regression of *DAM3* and *DAM4* relative expression with leaf area and petiole length.** Regression of *DAM3* and *DAM4* relative expression with leaf area and petiole length. The data of the start and induction phase (black) and the breaking phase (blue) were analyzed separately. Asterisks following the R^2^ values indicate significance: * P ≤ 0.05, ** P ≤ 0.01, *** P ≤ 0.001


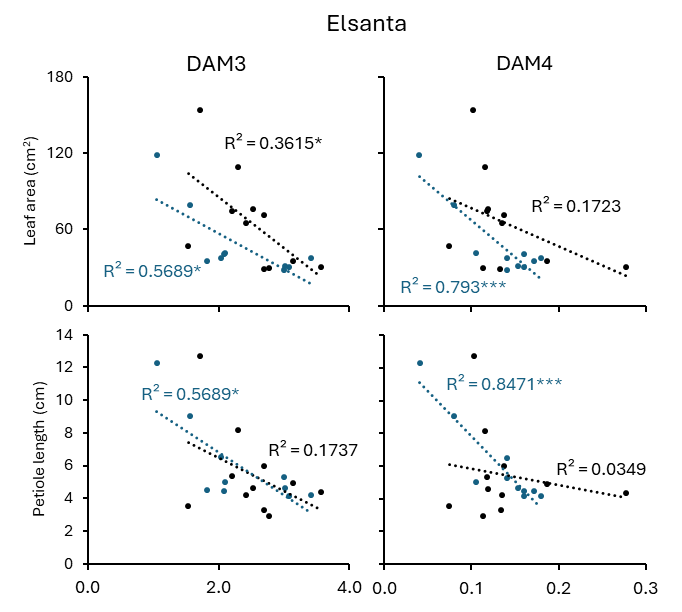

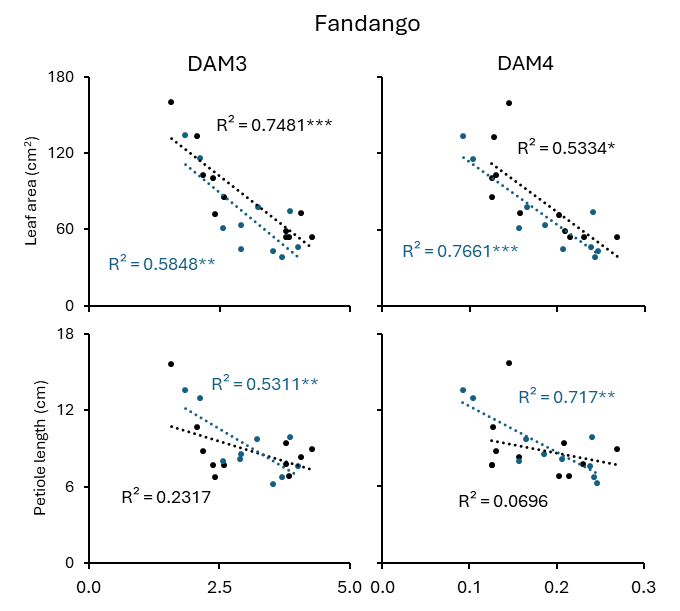

Supplement: Supplementary file 1 — Supplementary file1 (DOCX 123 KB) [file 425_2025_4799_MOESM1_ESM.docx]
